# Infantile Diabetes

**Published:** 1914-02

**Authors:** 


Infantile Diabetes. *Johns Hopkins Hosp. Bull.,* September, 1913. J. H. M. Knox has collected from the literature fifteen cases of diabetes in infants under a year. In three the disease was noted within a few days of birth. In three a grandparent or great-aunt had had diabetes. In six the disease seemed to have followed an injury to the central nervous system—in two a fall. In two an injury at birth was possible, as the labors were described as difficult. Three cases were associated with hydrocephalus and one with anencephalus. In three cases the symptoms began after the use of an unusual amount of sugar. One baby was fed on condensed milk; another was given 150 grams of sugar in addition to a litre of milk daily, and in the case reported about a litre of milk was given containing 10 per cent. malt soup. At least three cases ended in coma. Recovery took place in three. Thus the prognosis is grave, but not hopeless, except in a severe grade of the disease.

